# Encouraging adoption of green manure technology to produce clean rice product

**DOI:** 10.1038/s41598-023-35964-1

**Published:** 2023-05-29

**Authors:** Naser Valizadeh, Sara Jalilian, Zeynab Hallaj, Samira Esfandyari Bayat, Dariush Hayati, Khadijeh Bazrafkan, Nazanin Kianmehr, Morteza Akbari

**Affiliations:** 1 Department of Agricultural Extension and Education, School of Agriculture, Shiraz University, Shiraz, 7144165186 Iran; 2 Department of Agricultural Extension and Education, College of Agriculture, University of Tehran, Tehran, 7144165186 Iran; 3 Department of Agricultural Extension and Education, College of Agriculture, Tarbiat Modares University (TMU), Tehran, 1497713111 Iran; 4 Department of Technological Entrepreneurship, Faculty of Entrepreneurship, University of Tehran, Tehran, Iran

**Keywords:** Environmental sciences, Environmental social sciences

## Abstract

Green manure is used as an environmentally friendly technology to produce clean agricultural products. This technology not only helps reduce environmental and health concerns, but can also increase productivity. Green manure is especially needed in the production of paddy. Because rice as a strategic product is the main food of people in many countries of the world. Rice production using green manure can enable countries to develop and increase healthy production. However, the acceptance of this technology is low in many rice producing countries. In this regard, this study used an integrated and extended version of the theory of planned behavior to predict and encourage the adoption of green manure technology in Iran. To collect the required data, a cross-sectional survey was performed among Iranian rice growers and the results of hypothesis testing were analyzed using partial least squares-based structural equation modeling. The results revealed that moral norms of green manure, attitude towards green manure, perceived behavioral control on using green manure, and trialability of green manure have positive and significant effects on intention towards using green manure. In addition, bootstrap analysis showed that moral norms of green manure and trialability of green manure positively and significantly mediated the (indirect) effects of subjective norms towards application of green manure on intention towards using green manure. The results led to important practical and theoretical implications that could provide new insights for policy-makers, planners, and practitioners to develop and encourage the adoption of green manure technology to produce clean and healthy agricultural products.

## Introduction

In the past years and after the Green Revolution, many efforts have been made to increase food production, which has led to a significant increase in global food production. The results of this increase in production have been more in favor of developed countries and other regions of the world, especially underdeveloped countries have benefited less from this increase in production^1^. Accordingly, the agricultural research sector addresses issues related to food security, sustainable food production, environmental indicators, and socio-economic developments in rural-agricultural communities of underdeveloped and developing countries as a new agenda for agricultural production systems^2^. Today, agriculture is much more diverse than ever and is often combined with other activities. New agricultural knowledge produced by farmers, researchers, and private companies has created highly dynamic and complex knowledge networks^3^. However, the intertwined and specific relationship of agricultural systems with the environment distinguishes it from other economic sectors^4^. Because, an agricultural system is the result of the complex interaction of interdependent components such as water and soil, crops, labor and other resources in the environment^5,6^.

In addition, *technology* is one of the sources of agricultural production^1^. Increasing efforts in technology sector to increase production and diversify agricultural activities result from the continued growth of social expectations in the field of food security, animal welfare, bio-sustainability and pro-environmental production^7–9^. Sustainable farming and environmental-related technologies can be called Green Agriculture, which is an alternative approach to reduce the consumption of fossil fuels and minimize waste. In other words, the green agricultural systems, instead of using non-renewable and unstable inputs for agricultural production, use pro-environmental technologies such as green manure, biofertilizer, animal manure, etc. Since non-renewable resources endanger human, animal, and environmental health and cause climate change through global warming^10^. For example, overuse of nitrogen-based fertilizers in agricultural sector contributes to emissions nitrous oxide which is considered as one of the most important greenhouse gases. Leftover nitrogen that is not absorbed by agricultural products and plants, reacts with the soil. This reaction results in production of nitrous oxide. At a global level, agricultural sector accounts for about 80% of human-caused nitrous oxide emissions. This sector is also responsible for about 8–14% of all greenhouse gasses. In addition, ammonia compound, which is generally used in chemical fertilizers, also plays a role in climate change. Ammonia must be made under high pressure and at high temperature. In other words, it takes a lot of energy to produce it. Most of this energy comes from burning fossil fuels such as coal and methane gas. These fossil fuels lead to the production of carbon dioxide, which is the main cause of climate change. Today, ammonia production accounts for between 1 and 2% of carbon dioxide emissions worldwide^11,12^. Several definitions have been proposed for green technology. Soni^13^ states that green technologies are environmentally friendly technologies that address issues such as energy efficiency, recycling, increasing the use of renewable resources, reducing concerns about the safety and health, and so on. Green and environmental technologies produce less pollution and use all resources in a more sustainable way. These technologies recycle most of their waste and products and manage the remaining waste in a principled way with alternative technologies. In general, it can be understood that green agricultural technology includes renewable energy sources, biofertilizers, green manures, waste reduction methods, remediators of environmental pollutants, sewage treatment, waste water recycling, and improving the agricultural systems, which have positive effects on environmental decisions^14^.

Green manure technology refers to plants that have already been uprooted and are often already placed under the soil. These dying plants are cover crops that are grown mainly to add nutrients and organic matter to the soil. Typically, a plant used as a green manure is grown for a period of time and then plowed and incorporated into the soil while it is still green or shortly after flowering. Green manure products are generally associated with organic farming and are considered essential for ship systems that are supposed to be stable for many years^15^. In this study, the use of green manure is considered as a “technology”. Technology is a set of processes, methods, techniques, tools, equipment, machinery, and skills by which a product is made or a service is provided^16^. There are two reasons why green manure can be considered as a technology. First, as defined by Januszewski and Molenda^16^, technologies are not just hardware tools or machines. Rather, the inputs by which a service is provided are also technologies. In this study, the use of green manure is considered as a method through which healthy rice can be produced. Second, according to Fathian and Mahdavi-Noor^17^, if there is a hard-soft technology that can transform renewable and non-renewable natural resources into usable services for users, it can also be called technology. Considering that green manure is a technique that leads to the use of plant residuals to produce a green product, it can be considered as a technology.

Green manures are made from a combination of plant materials (either freshly cut weeds or rotation crop debris) and added to the soil while they are still green^18^. Different types of green manures (such as *Astragalus sinicus* L., *Vicia villosa* Roth., and *Medicago sativa* L.) have different functions and applications in environmental protection, sustainable development, and agricultural economic development. For example, green manures help increase humus in the soil, carbon sequestration, and improve soil fertility^19^. Crop residues and green manures release their nutrients after decomposition in soil^18^. This process increases the content of soil organic matter (humus, carbon, and nitrogen) and improves the soil-forming structure. Green manures maintain the nutrient cycle in the agricultural ecosystem and enhance the biomass and activities of soil microorganisms^19^. Also, these manures help control weeds, pests and plant diseases, and reduce soil erosion^20^. Therefore, green manure products and technologies play an important role in reducing the use of chemical fertilizers^21^.

Another point that is very important in the discussion of the production and consumption of green products and their effects is the circular premium that the consumers of the products accept when purchasing^22^. Conceptually, circular premium is defined as the consumers’ willingness to pay more for circular products. Consumers may be willing to accept that a circular price of the rice (i.e., the price required for a product obtained with a completely sustainable approach) is different from the normal price (i.e., the price they currently pay for the products being produced with chemical fertilizers)^23^. The review of research literature in this field shows that the circular premium has positive impacts on the development of the circular economy and the production of sustainable products. For example, Colasante and D'Adamo^24^ and Appolloni et al.^25^ introduce the concept of “green circular premium” and state that strategies like green circular premium and sustainability certification can create a sustainable competitive advantage in today's uncertain world for mature industries and producers. In other words, one of the impacts of the green circular premium is that it makes the producers not consider innovation in production and process as the only strategies to maintain and increase income and competitiveness^23^.

Although there is still no general agreement on the concept of clean agricultural products among experts and researchers, in the present study, clean rice product refers to the rice product that farmers did not use chemical fertilizers for its production. On the other hand, they have used rice plant residues as the manure to increase their production. In some cases, researchers use the term “green fertilizer” instead of “green manure”. But it should be emphasized that there are six basic differences between fertilizers and green manures. First, green manures are obtained naturally by the decomposition of dead plants and residues. However, fertilizers are chemical substances and are not typically natural. Second, although manures are not very rich in nutrients, fertilizers are rich in soil nutrients like nitrogen, phosphorous, and potassium. Third, despite fertilizers that are easily absorbed by plants. manures are slowly absorbed. Fourth, manures provide a lot of humus to the soil; but fertilizers do not provide any humus to the soil. Fifth, manures are prepared naturally in the fields. However, fertilizers are prepared in the factories. Sixth, manures do not adversely affect the plant or the soil if supplied in large quantities. That is while fertilizers adversely affect the soil and the plant if supplied in large quantities^26,27^.

Although planting green manure in fallow croplands in winter can have a variety of economic and environmental benefits, including carbon capture and sequestration, soil retention, sandstorm prevention, water retention, and provision of habitat for biodiversity and the government enthusiastically supports the planting of green manures, the implementation of this action is slow. Furthermore, it should be mentioned that increasing the cost of production and planting green manures has reduced the willingness of farmers to adopt green manures^28^. That is while different stakeholders’ engagement in food production process is of great importance. Stakeholders in the food production chains are very extensive. These stakeholders can range from individual consumers of different food products and industry bodies to primary producers such as farmers. The engagement of these stakeholders is necessary to produce green products. In a study that D'Adamo^29^ conducted to enable stakeholder engagement for sustainability reporting in the food industry, concluded that stakeholder engagement is an order winner for sustainable strategies in the food (pasta) industry. Leonidou et al.^30^ and Shams^31^ in their studies on the role of stakeholders’ engagement in the food industry claim that if this process is managed correctly, it can make the food industry work more effective in line with sustainable principles. Considering stakeholder theory, there are differences between internal and external stakeholders in terms of role-playing in the food industry^32–34^. However, according to Wolf^35^, at the food supply chain level, the role and effect of external stakeholders seems complex. Greenwood^36^ and Giacomarra et al.^37^ concluded that stakeholder engagement plays a key role in identifying key stakeholders and identifying unproductive interactions. In other words, in the stakeholder engagement process, the companies and industries can determine which stakeholder(s) to cooperate with and how to cooperate. Also, Greenwood introduces “knowledge sharing” as the main role of stakeholder participation. Kazadi^38^ also states that stakeholders' engagement in industries such as the food industry can strengthen the collaborative production of knowledge. This has a significant role in increasing competitiveness, motivating work, and encouraging and developing food innovations. Ghassim and Bogers^39,40^ call this role of stakeholder engagement “accumulation of valuable capabilities”.

Subsidy policies for green manure planting are still under research and development. There is currently no subsidy policy and the cost of planting green manure is high in terms of economic outcomes. In addition, there are no formal or sufficient incentives to encourage farmers to voluntarily plant green manure^19^. In some cases, factors such as lack of operational conditions (access to credit, seeds, machinery, etc.) are limiting factors in the development and use of green manure by farmers^39,40^. Another obstacle that worries farmers is the cultivation and occupation of the farm with crops that do not lead to an immediate return on capital and profits in the short term. Although long-term and medium-term benefits for commercial crops and soil should be considered, in many cases the poor economic strength of farmers prevents them from paying attention to long-term benefits. Limitations related to the size of agricultural lands, labor costs, management of some species used in green manure^41^, lack of cost-effective and quality seeds, lack of adequate knowledge, limited capital, land competition^20^, lack of proper regulatory framework, manpower shortages, performance uncertainties, and financial, political, cultural, and legal issues^13^ are of the most important constraining factors in the adoption and application of green manure technology by farmers. In some cases, these obstacles have caused the development of the use of chemical fertilizers in countries like Iran. A review of the statistical data of the Ministry of Agriculture of Iran shows that the average use of chemical fertilizers in Iran is about 70 kg per hectare^42^.

In addition to the issues and problems mentioned above, it should be emphasized that the lack of sufficient knowledge of policy-makers, decision-makers, managers, and practitioners about the determinants, including farmers' intentions to use green manure is another major problem leading to failure in the transfer of green manure technology^43^. In this regard, the main objective of the present study was to analyze the intentions of Iranian rice farmers in to adopt green manure technology. To achieve this objective, following some sub-objectives were defined:

1. Developing a theoretical framework based on the theory of planned behavior;
2. Running the measurement model of the framework to assess the outer model’s reliability and validity;
3. Running the structural model of the framework to test the hypotheses and assess the inner model’s reliability and validity; and
4. Interpreting the results and presenting some theoretical and practical policy implications.

## Theoretical background and formulation of framework

The theory of planned behavior (TPB) is known as one of the most practical theories to explain the behavioral intentions of individuals, which is mediated by three key variables^44–46^. Intention in this theory is defined as an action-oriented future behavior that may occur in the very near future^47,48^. For example, the intention to adopt green manure technology refers to a series of future-oriented behaviors that farmers may take in the very near future to apply this technology^43^. The three main antecedents or determinants of adoption in TPB include attitude towards green manure, perceived behavioral control on using green manure, and subjective norms towards application of green manure^43,49–53^. Attitude refers to an individual’s evaluation about a specific behavior in terms of the desirability or un-desirability^54^. In this study, attitude towards green manure refers to farmers' evaluation of the desirability or un-desirability of the practice of using green manure. Subjective norms refer to out-of-person control interactions that direct his/her behavior^55,56^. In other words, subjective norms refer to the views of others about whether or not a person should perform a particular behavior^47,48^. In present study, subjective norms towards application of green manure measures the impact of others on the green manure technology acceptance behavior. Perceived behavioral control also represents the perceived difficulty or ease in performing a behavior^51^. Some researcher including Valizadeh et al.^56^ consider this variable as human agency. Perceived behavioral control on using green manure is based on the key to how far farmers think it is difficult or easy for them to adopt green manure technology. Although the predictive power of TPB in explaining different behavioral intentions has been proven in various studies (see^48,50,57,58^), it is still criticized^58^. These weaknesses have led to the development of this theory among researchers in various scientific fields, especially environmental psychology, has become an emerging discourse^59^.

Regarding the weaknesses of TPB, Pradhananga et al.^60^ and Haji et al.^59^ stated that this theory considers individual behavior as a rational behavior. In other words, the reason for the behavior in this theory goes back to the personal/private-sphere interests of individuals^61,62^. Stern^63^ argues, however, that people's behaviors or behavioral intentions do not always stem from their personal interests. In some situations, factors such as moral and altruistic considerations can also guide individuals' behavior^64^. Such critiques have led proponents of moral theories (value-belief-norms theory and norm activation theory) to see the extension of TPB theory using moral norms as an undeniable necessity. Therefore, in this study, the variable of moral norms of green manure was incorporated into TPB as a new variable. Moral norms of green manure refer to the sense of moral (personal) responsibility of farmers in using green manure on farms. In other words, farmers consider the use of green manure as a moral responsibility, which prevents the destruction of the environment and results in sustainability of agricultural activities.

**Figure 1:**
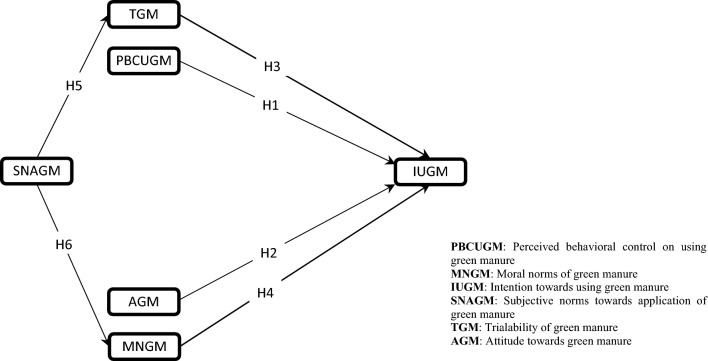
Theoretical framework of the study.

In development process of TPB, other new theories and perspectives have been proposed. In particular, Innovation diffusion Theory (IDT) emphasizes the importance of innovation features. The most important determinants of technology acceptance and non-acceptance behavior in this theory include comparative advantage, compatibility, complexity, trialability, and visibility^65^. However, it is noteworthy that not all of the features in this theory can be added to TPB. Because features such as comparative advantage, compatibility, complexity, and visibility are reflected by different names in the TPB. The complexity variable, for example, is called ease of use in TPB. In addition, the attitude in TPB itself includes all the factors of comparative advantage, compatibility, and visibility. Accordingly, in present study, trialability of green manure was the only variable added to TPB from IDT theory. Finally, the extended version of TPB was presented as Fig. 1. The direction of the arrows in this figure shows how independent variable affects dependent variables. From an agricultural perspective, trialability means the extent to which the technology can be assessed on a small scale before it can be extensively implemented. Trialability of green manure reduces risk by providing useful information about hazards and technology uncertainty^43^.

Regarding the relationships of variables in Fig. 1, it should be emphasized that according to the initial version of TPB, the variables attitude towards green manure and perceived behavioral control on using green manure have direct effects on intention towards using green manure. In this regard, these two variables were directly affected intention towards using green manure. As mentioned earlier, attitude towards green manure conceptually refers to farmers' evaluation of the desirability or un-desirability of the practice of using green manure. However, perceived behavioral control on using green manure is based on the key to how far farmers think it is difficult or easy for them to adopt green manure technology. From the definitions of these two variables, it can be understood that perceived behavioral control on using green manure emphasizes more on the evaluation of self-efficacy in the use of green manure. At the same time, the attitude is focused on the person's beliefs about green manure and his/her evaluation of its positive and negative consequences. Therefore, it is possible to examine the effects of these two variables on intention separately and interpret their relationship with intention in the form of two separate hypotheses. In addition, according to the assumptions of moral approaches to environmentalist behaviors, moral norms of green manure also have a direct effect on intention. IDT also introduces trialability as one of the direct predictors of intention towards using green manure. However, in this study, we hypothesized that subjective norms towards application of green manure indirectly through moral norms and trialability activates intention. In other words, moral norms and trialability mediate the effect of subjective norms on intention. Finally, the research hypotheses were configured as follows:

1. Perceived behavioral control on using green manure positively and significantly will affect intention towards using green manure;
2. Attitude towards green manure positively and significantly will affect intention towards using green manure;
3. Trialability of green manure positively and significantly will affect intention towards using green manure;
4. Moral norms of green manure positively and significantly will affect intention towards using green manure;
5. Trialability of green manure positively and significantly mediate the effect of subjective norms towards application of green manure on intention; and
6. Moral norms of green manure positively and significantly mediate the effect of subjective norms towards application of green manure on intention.

## Methodology

### Study context

This study was conducted in Fars province of Iran, which is located in the southwest of the country. With a population of nearly 5 million people, this province is one of the most populous provinces in Iran. Having the province with fertile soils and suitable climate, has led to the development of agricultural activities in it. In other words, fertile soils have made this province one of the centers of production of strategic products for Iran from wheat, barley, rice, and oilseeds. This has made the agricultural sector of Fars province one of the pillars of food security in a country like Iran, which has been under international sanctions for years. One of the leading products for Iran and its growing population is rice. More interestingly, despite the strategic importance of this product for Iran, its production is possible in many limited parts of the country. Meanwhile, Fars province is the most important producer of rice after the northern provinces of the country (Mazandan, Guilan, and Golestan). However, rice production in this province has been associated with many problems in recent decades. Factors such as not using new agricultural methods, lack of water resources, and lack of access to sufficient credit are new obstacles to sustainable production of rice in the province. The synergistic effect of these issues has also shown itself in the form of some rebound effects, which has caused the most damage to the environment of the province. In this regard, many experts believe that in the process of rice production in Fars province, sustainable/clean production methods should be used to minimize the impact of environmental impacts. One of the strategies proposed to achieve this goal is to encourage farmers to use green manures. Unfortunately, evaluations have shown that the use of this technology is low among rice farmers. In addition, no study has been conducted on the determinants of farmers' willingness to use green manures on farms. This factor has made the acceptance of green manures as a research priority for agricultural executive organizations in the province. In this regard, the aim of this study was to identify and analyze the socio-psychological mechanisms of acceptance of green manure technology among the rice farmers.

### Data collection and sampling

We used a questionnaire-based survey to collect the information. But before entering the main survey stage, four important steps were taken. In the first step, the prepared questionnaire was given to a panel of experts in environmental psychology, agricultural extension and education, and practitioners of new agricultural technology development. This was done by the first and third authors. The panel of experts presented their views on the face and content validity of the questionnaire. Therefore, items that may not be related to the measurement of the variables were removed from the questionnaire. Also, some questions (items) were modified to adapt to the participant's understanding. In the second step, the necessary coordination was done with the local leaders and village heads (managers); so that the first and third authors could make initial visits and evaluations of the rice fields of the province. In the process, some initial talks were even held with some farmers to discuss their issues and problems with the use of technologies such as green manures. In the third step, a number of questionnaires were filled out by farmers as pilots. Completion of these questionnaires allowed researchers to be informed of the time required to complete the questionnaires, incomprehensible words or items, barriers, and drivers of an effective communication (while collecting data) with farmers. In the fourth step, the total number of rice farmers in the province was inquired from the Agricultural Jihad Organization of Fars Province. According to the information provided by this organization, there were 10,158 rice farmers in Fars province. Therefore, 375 of them were selected as a sample through Krejcie and Morgan sampling table. According to Agricultural Jihad Organization of Fars Province, most of the rice farmers have a traditional farming system and do not mechanize rice cultivation. In addition, in terms of gender, most of the farmers are men, but women also help men in harvesting stage. Because the rice production process in Fars province is generally traditional, usually the young people are less willing to continue their father's job. Of course, it should be noted that in cases where young farmers decide to continue their father's job, they try to change the rice production process from traditional to mechanized. A data collection team was used to collect the required information. The team consisted of six skilled researchers led by the first author. The members of the data collection team had two special characteristics. First, they all had high experience in collecting and processing survey data. Therefore, they were fully familiar with the techniques of gathering information and communicating with the participants in this type of studies. Second, five of them (except the first author) were natives of the study area. This would help them collect more reliable data. Systematic random sampling method was used to select the samples. Systematic random sampling is a transformed simple random sampling method. In this sampling method, the distance and order of sampling is obtained by dividing the population size by the sample size. This means that each person is systematically selected from a non-ranked list based on regular intervals and in a specific order. A systematic sample is generally distributed more evenly throughout the population, resulting in more information (than simple random sampling with the same population size) about the population. Therefore, due to the characteristic of obtaining more information per unit cost, this method is very suitable for studies and surveys that work with budget constraints. Systematic sampling is often easier to implement in structure, execution, comparison, and understanding than simple randomization. Hence this sampling method is more popular among researchers. It is worth mentioning that the possibility of error by the questioner in this method is reduced.

### Statement

All interviewees were informed about data protection issues by the enumerators and gave their consent orally at the beginning of each interview. Informed consent was obtained from all individual participants included in the study. All materials and methods are performed in accordance with the instructions and regulations and this research has been approved by a committee at Shiraz University, Iran. This research has been approved by an institutional review board at Shiraz University, Iran. All procedures performed in studies involving human participants were in accordance with the ethical standards of the institutional research committee and with the 1964 Helsinki declaration and its later amendments or comparable ethical standards.

### Measures

This study is part of a larger project on the willingness to adopt green manure technology in Fars Province, Iran. Participants' responses to each of the measures and questions were used to address the questions and achieve the objectives of the present study. The measures used included some of the key variables of technology adoption pattern and innovation dissemination theory that were used to construct the theoretical framework of the research (Fig. 1). Based on the conceptual framework of the research, the main measures included perceived behavioral control on using green manure, moral norms of green manure, intention towards using green manure, subjective norms towards application of green manure, trialability of green manure, and attitude towards green manure.

To measure perceived behavioral control on using green manure, moral norms of green manure, intention towards using green manure, subjective norms towards application of green manure, trialability of green manure, and attitude towards green manure we used three, four, three, three, three, and six items, respectively, all of which were adapted from previous research studies on conservation behaviors. Perceived behavioral control on using green manure items were adapted from Savari et al.^64^, Bagheri et al.^48^, and Haji et al.^59^, moral norms of green manure items were adapted from Yazdanpanah et al.^66^ and Stern^63^, intention towards using green manure items were adapted self-developed, subjective norms towards application of green manure items were adapted from Savari et al.^64^ and Mancha and Yoder^54^, trialability of green manure items were adapted from Adnan et al.^43^ and Haji et al.^59^, and attitude towards green manure items were self-developed. It should be noted that all of these constructs were measured using a five-point Likert scale (strongly disagree: 1 strongly agree: 5). The measuring items of each of these constructs have been presented in Table 1.

**Table 1:** Survey items and Cronbach's alpha coefficients.

Var	No	Items	Source*
	Perceived behavioral control on using green manure: (α = 0.83)		
Perceived behavioral control on using green manure	1	It is easy for me to use green manure technology	1, 2, and 3
	2	It is easy to learn how to process and use green manure technology	
	3	Any rice grower in this area can use green manure technology	
	4	Processing and using green manure require a lot of effort	
	Moral norms of green manure: (α = 0.76)		
Moral norms of green manure	1	Helping to protect the soil with green manure is a moral responsibility for us farmers	4 and 5
	2	Helping control pests and diseases by using green manure is a moral responsibility for us farmers	
	3	Using green manure instead of chemical manures is a moral and public-sphere act	
	4	By using green manure, we can fulfill our moral responsibility to protect natural resources for future generations	
	Intention towards using green manure: (α = 0.82)		
Intention towards using green manure	1	I intend to recommend green manure technology to others	Self-developed
	2	I plan to use green manure technology in my rice field	
	3	I want to learn the skills needed to process and use green manure	
	4	I am ready to accept the challenges of using green manure technology	
	Subjective norms towards application of green manure: (α = 0.71)		
Subjective norms towards application of green manure	1	My acquaintances and people around me think that I should use green manure technology	1 and 6
	2	The use of green manure technology in the rice field leads to my approval by those around me	
	3	To the satisfaction of my acquaintances and those around me, I try to use green manure technology on the farm	
	Trialability of green manure: (α = 0.71)		
Trialability of green manure	1	I can try green manure before deciding	3 and 7
	2	I can do green manure technology in a small part of the rice field	
	3	The benefits and technological effects of green manure can be examined with a simple small-scale experiment	
	Attitude towards green manure:(α = 0.71)		
Attitude towards green manure	1	I think it is very important to promote the use of green manure in rice fields	Self-developed
	2	Using green manure instead of chemical manure is a rational task	
	3	The use of green manure technology in rice farms should be further developed	
	4	Due to the (economic and environmental) characteristics of green manure technology, we farmers have to use it	
	5	The effect of using green manure technology on different dimensions of crop production should be evaluated in the long run	
	6	I like the idea of using green manure on rice fields	

*1: Savari et al.^64^, 2: Bagheri et al.^49^, 3: Haji et al.^60^, 4: Yazdanpanah et al.^67^, 5: Stern^63^, 6: Mancha and Yoder^55^, and 7: Adnan et al.^44^.

### Reliability and validity of measures

Internal consistency reliability of the measures was evaluated using Cronbach's alpha coefficients and composite reliability (CR). The validity of the measures was assessed using convergent and divergent validity assessment methods. AVE and Fornell-Larker criteria were used to evaluate the convergent and divergent validity, respectively. The results of all indices are discussed in the research results section.

### Data analysis

Data analysis was performed using Structural Equation Modeling (SEM) based on Partial Least Square (PLS based SEM). There were several major justifications for using this method. First, one of the objectives of the study was to predict the intention of the farmers to use green manure. As a result, an attempt should be made to use a method to maximize the variance of explanation by latent internal variables. PLS based SEM was one of the best ways to meet such a goal. Second, according to Hair et al.^67^, PLS based SEM is an efficient method for implementing and interpreting integrated models. Third, this method and the software used for it are much more user-friendly than other methods and software. PLS based SEM consists of two estimation processes that include evaluation of the measurement model and structural model. In fact, the measurement model tries to determine the role of each indicator in explaining its corresponding latent measure. In the structural model, however, the indicators are not the basis for evaluation. In other words, the structural model involves examining the relationships between latent variables in a theoretical framework. In the structural model, the predictive ability of the hypothetical model is examined^67^.

## Results

### Measurement model

Internal consistency reliability tests the hypothesis that the indicators proposed to measure a variable have similar results^68^. The rule of thumb for proper reliability is that Cronbach's alpha and the CR of measures should be greater than 0.7. Based on the results of Table 2, all values related to these two indicators of internal consistency reliability were accepted. The only exception was perceived behavioral control on using green manure, with an alpha value of 0.555. Although this value of Cronbach's alpha may be small according to many statistical sources, there are researchers (see^69,70^) who consider alpha values above 0.5 to be an acceptable value for internal consistency reliability. Convergent validity is an evaluation criterion that shows the degree of correlation of one indicator with other indicators of a theoretical construct^68^. For this purpose, the values of loading factors and AVE index are usually used. The values of the AVE index were presented in Table 2 and the values of the loading factors together with their corresponding T-statistics were presented in Table 3. Considering that the values of these two indices for all indicators and measures were higher than 0.7 and 0.5, respectively, we concluded that convergent validity was confirmed. The only exception to the loading factor values was the first item of perceived behavioral control on using green manure. Due to the fact that this indicator showed a factor loading of 0.671 (lower than 0.7), we removed it from the model. It should be noted that the significance/non-significance and the result of the hypotheses related to the significance of the loading factors of the indicators were also presented in Table 3.

**Table 2:** Measurement items and indicators of model fit.

Variable	Cronbach’s alpha	Composite reliability	Average variance extracted (AVE)	*P* values
Intention towards using green manure	0.930	0.955	0.877	0.001
Attitude towards green manure	0.910	0.930	0.689	0.001
Moral norms of green manure	0.907	0.935	0.783	0.001
Perceived behavioral control on using green manure	0.555	0.768	0.526	0.001
Subjective norms towards application of green manure	0.812	0.888	0.727	0.001
Trialability of green manure	0.772	0.868	0.686	0.001

Acceptable values for the reported indices: Alpha > 0.7; *p* < 0.01; CR > 0.7; and AVE > 0.5.

**Table 3:** Measurement items, loading factors and T-value of the model.

Factors	Indicators	Loading factor	T-value	Significant	Result
Perceived behavioral control on using green manure	Item2	0.671	10.802	0.001	Accepted
	Item3	0.715	14.176	0.001	Accepted
	Item4	0.785	21.883	0.001	Accepted
Trialability of green manure	Item1	0.805	36.238	0.001	Accepted
	Item2	0.834	36.327	0.001	Accepted
	Item3	0.846	40.115	0.001	Accepted
Subjective norms towards application of green manure	Item1	0.836	32.703	0.001	Accepted
	Item2	0.873	42.296	0.001	Accepted
	Item3	0.843	36.667	0.001	Accepted
Moral norms of green manure	Item1	0.894	42.056	0.001	Accepted
	Item2	0.910	74.150	0.001	Accepted
	Item3	0.894	54.993	0.001	Accepted
	Item4	0.840	33.131	0.001	Accepted
Attitude towards green manure	Item1	0.853	36.819	0.001	Accepted
	Item2	0.837	39.298	0.001	Accepted
	Item3	0.831	36.581	0.001	Accepted
	Item4	0.824	37.162	0.001	Accepted
	Item5	0.822	37.606	0.001	Accepted
	Item6	0.814	32.353	0.001	Accepted
Intention towards using green manure	Item2	0.927	72.229	0.001	Accepted
	Item3	0.923	61.035	0.001	Accepted
	Item4	0.960	134.179	0.001	Accepted

Acceptable values for the reported indices: all loadings > 0.7; *p* < 0.01; CR > 0.7; and AVE > 0.5; T value > ±1.9.

Discriminant or divergent validity indicates how different a theoretical construct is from other structures within a conceptual framework^68^. As mentioned in the methodology section, in the present study, the Fornell–Larker criterion was used to assess divergent validity. According to the results reported in Table 4, the values in the matrix diameter are greater than the values in their corresponding columns. This result shows that the research tool used in this study had a suitable divergent validity. In other words, all structures are practically different from other framework structures.

**Table 4:** Assessment of the discriminant.

Variable	Validity					
	1	2	3	4	5	6
Intention towards using green manure (1)	**0.936**					
Attitude towards green manure (2)	0.864	**0.830**	–	–	–	–
Moral norms of green manure (3)	0.882	0.933	**0.885**	–	–	–
Perceived behavioral control on using green manure (4)	0.638	0.457	0.525	**0.725**	–	–
Subjective norms towards application of green manure (5)	0.829	0.844	0.817	0.456	**0.852**	–
Trialability of green manure (6)	0.787	0.764	0.763	0.422	0.795	**0.828**

Significant values are in bold.

### Structural model and testing the hypotheses

**Figure 2:**
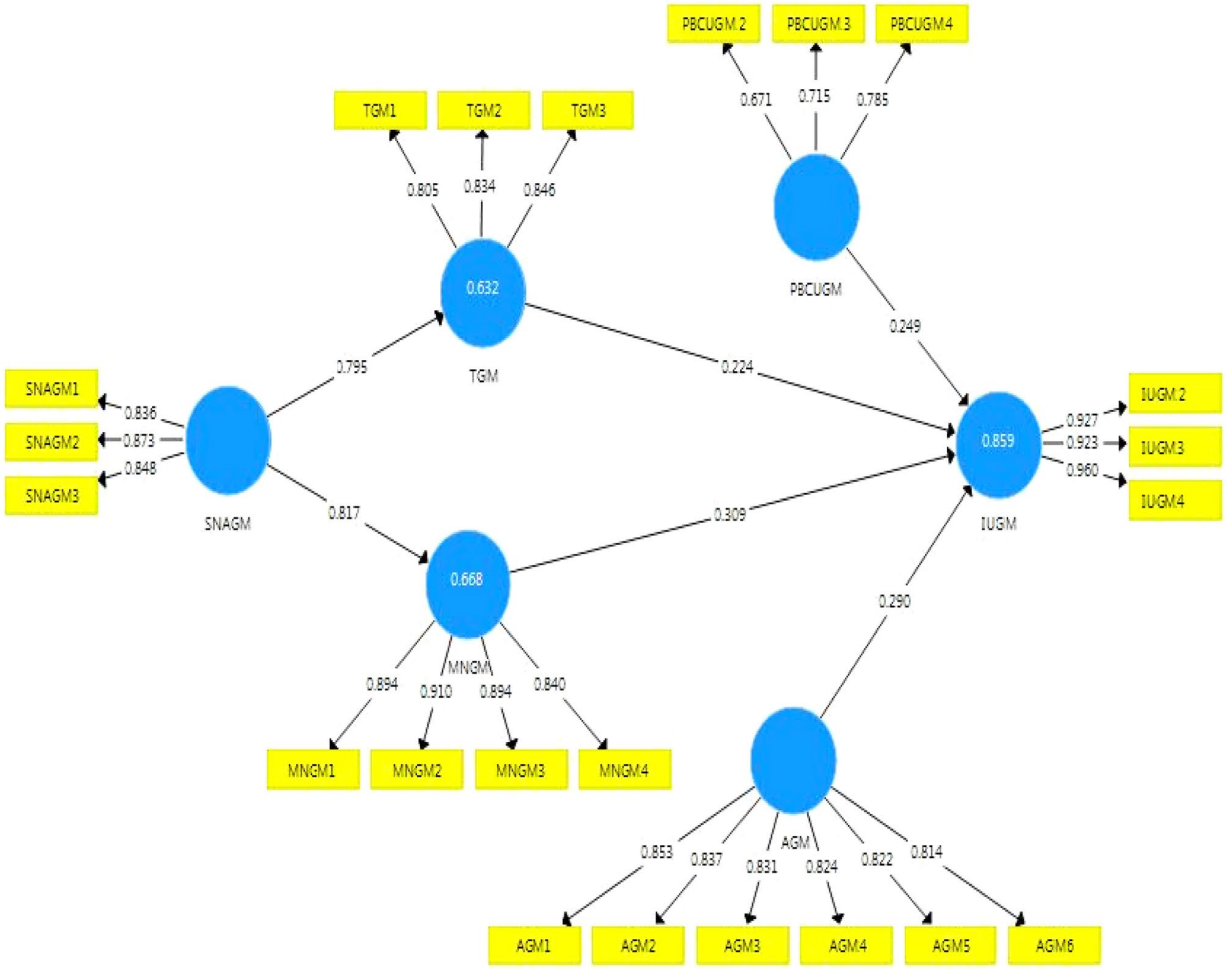
The PLS based SEM model with standardized path coefficients.

In order to understand the significance of the hypothesized paths, we employed the structural model (Fig. 2). In addition, the structural model helps to determine the predictive power of the model. In order to test the significance of the hypotheses, the bootstrapping method was used. The latent variables in the structural model play a key role in identifying the explanatory power of the main independent variable. Therefore, coefficients of determination (R^2^) of endogenous variables of the structural model were used to judge the predictive power of the model. According to Hair et al.^68^, the values above 0.75 for R^2^ are considered appropriate values in a structural model. In the present study, the value of R^2^ for intention towards using green manure was 0.86, indicating that the model presented has a good predictive ability (Table 5). In other words, the exogenous structures were able to well explain the dependent intention towards using green manure.

**Table 5:** Estimated effects on intention.

Hypothesis	Direct effects		Indirect effects		Total effect	R^2^	Q^2^	Result
	T	Beta	T	Beta				
H_1_: Perceived behavioral control → Intention	7.443**	0.249	–	–	0.249	0.86	0.73	Accepted
H_2_: attitude → Intention	3.675**	0.290	–	–	0.290			Accepted
H_3_: Trialability → Intention	5.589**	0.224	–	–	0.224			Accepted
H_4_: Moral norms → Intention	3.303**	0.309	–	–	0.309			Accepted
H_5_: Subjective norms → Trialability → Intention	–	–	5.327**	0.179	0179			Accepted
H_6_: Subjective norms → Moral norms → Intention	–	–	3.128**	0.253	0.253			Accepted

ns = Not significant; *p < 0.05; **p < 0.01.

The results of the analysis of direct effects on intention towards using green manure in the structural model (Table 5) showed that perceived behavioral control on using green manure positively and significantly affected intention towards using green manure (Beta = 0.249; T = 7.433). This result demonstrates that the first hypothesis of the research has been confirmed. The results of testing the effect of attitude towards green manure on intention towards using green manure (second hypothesis) also revealed a positive and significant effect (Beta = 0.290; T = 3.675). The third hypothesis tested the effect of trialability of green manure on intention towards using green manure. The results of hypothesis testing indicated that trialability of green manure has a positive and significant effect on intention towards using green manure (Beta = 0.224; T = 5.589). Testing the effect of moral norms of green manure on intention towards using green manure was the fourth and final hypothesis to examine the direct effects on intention. Based on the results of the hypothesis test, moral norms of green manure positively and significantly affected intention towards using green manure (Beta = 0.309; T = 3.303). Comparison of the results of direct effect scores shows that among the independent variables, moral norms and attitude towards green manure have the highest ability to predict intention towards using green manure, respectively (Table 5).

The mediated effects of subjective norms towards application of green manure on intention were investigated in the form of hypotheses five and six (Table 5). The results of testing the fifth hypothesis revealed that the effect of subjective norms on intention towards using green manure is positive and significant (Beta = 0.179; T = 5.327). This result suggests that trialability of green manure can mediate the effect of subjective norms on intention. Based on the results of testing the sixth hypothesis, the effect of subjective norms towards application of green manure on intention was positive and significant. This result indicates that moral norms of green manure can also mediate the effect of subjective norms on intention (Beta = 0.253; T = 3.128).

Also, subjective norms towards application of green manure in the role of exogenous structures was able to predict 0.64 and 0.67% of the variance changes of endogenous structures trialability and moral norms of green manure, respectively. The R^2^ index actually indicates the degree to which the dependent variable is explained by independent variables. Acceptable values for this index are values between 0 to 1. The larger the value of this index, the higher the accuracy of the prediction. Hair et al.^71^ state that the values 0.19, 0.33, and 0.67 can be considered as weak, medium, and strong values, respectively. Examination of the values obtained for the present study revealed that R^2^ is at a desirable and acceptable level. In addition to evaluating the magnitude of R^2^, the value of Q^2^ index was also examined. This index is one of the other fit indices in SMART PLS that is used to check the predictive relevance of the model. If the value of this index is more than 0 for a latent structure, it can be concluded that the predictive relevance of the model is appropriate for that structure. However, if the value of this index is zero and less, the path model has no predictive relevance with the given structure. The values 0.02, 0.15, and 0.35 are considered small, medium and large predictive relevance values in a model, respectively. In the study, the values of Q^2^ for trialability of green manure and moral norms of green manure as the endogenous structures were 0.416 and 0.504, respectively (Table 5), so it can be concluded that the path model for trialability of green manure and moral norms of green manure as the endogenous structures is also appropriate (Table 5). In addition, the total Q^2^ value for the intention towards using green manure was 0.725, which indicates a good and high predictive relevance.

Examination of goodness indices (Table 6) of the model showed that in general, the presented or estimated model has a good fit. The squared values of the squared Euclidean distance (d_ULS_) and the geodesic distance (d_G_) were significant at the level of 0.05 error. This result demonstrate that the model estimation is done efficiently. The value of the Standardized Root Mean Square Residual (SRMR) index was 0.107, which indicates that the measurement error in the correlation matrix is acceptable. The root mean square error correlation (RMS_theta_) index is used to distinguish ill-specified models from well-specified models^72^. If the value of this index is greater than or equal to 0.12, it can be concluded that the model presented and tested is a well-specified model. In the present study, the value of this index was 0.270, which indicates an acceptable value.

**Table 6:** Goodness of the fit indices for the research model.

Fit index	SRMR	d_ULS	d_G	NFI	RMS Theta
Recommended value	< 0.1	> 0.05	> 0.05	> 0.80	≥ 0.12
Estimated value	0.107	2.918	2.194	0.612	0.270

## Discussion and policy implications

The results showed that moral norms of green manure had a positive and significant effect on intention towards using green manure and this variable was the strongest predictor of intention. In other words, the higher the moral norms of green manure among farmers, the more they will be inclined to use green manure in rice cultivation. This suggests that encouraging farmers to use green manure by evoking their moral and personal responsibilities can have a significant impact on improving their intention to use green manure technology. Similar results can be found among the results of researchers such as Zhang et al.^73^, Savari et al.^64^, Yazdanpanah et al.^66^, Alzaidi and Iyanna^74^, and Gholamrezai et al.^75^. This result shows that the use of tools and strategies to strengthen the moral norms can still be one of the solutions to the problem of reluctance or unwillingness of farmers to use green manure. In this regard, it is recommended that in the first step to encourage intention towards using green manure, the sense of moral responsibility of farmers to use green manure be strengthened. For this purpose, it is necessary to use different strategies. One of the most important strategies for developing moral norms regarding the use of green manure is to reward farmers who use this technology in their agricultural operations. The fact is that due to the lack of attention to the moral and responsible activities of farmers and the lack of encouragement of these behaviors, such feelings no longer appear in many of them. But it is possible to use encouragement to activate a sense of moral responsibility in them, and therefore help encourage the desire to use green manure technology. The second strategy that can be used to develop moral norms in farmers is self-education and self-judging. In this way, technology transfer officials and practitioners in agricultural communities must first try to teach farmers how to evaluate an agricultural activity morally. To this end, they can introduce criteria for the immorality of these activities. For example, if doing a particular agricultural activity endangers the health of other farmers, it can be considered immoral. In the next stage, farmers must work together collectively to define criteria and to judge and evaluate their agricultural activities. The results of testing the sixth hypothesis also emphasize the importance and necessity of using these strategies. Because, testing this hypothesis revealed that moral norms mediate the relationship between subjective norms in the use of green manure technology and intention. In other words, increasing or decreasing moral norms in the agricultural community can greatly increase or decrease the effect of subjective norms on intention towards using green manure.

The results showed that attitude towards green manure is the second most powerful construct affecting intention towards using green manure. In other words, intention towards using green manure can be improved by forming a favorable attitude towards the use of green manure technology. This result is in line with the results of Hua and Wang^57^, Yarimoglu and Gunay^50^, Bagheri et al.^48^, and Aboelmaged^58^. Attitude towards green manure is important from several aspects, which makes it necessary to focus on it in research related to the use of green manure or other pro-environmental behaviors. First, attitude is closely related to other psychological variables such as values, beliefs, norms, etc. in individuals’ memory. In this regard, creating a favorable attitude towards a pro-environmental technology such as green manure can help strengthen other psychological factors predicting intentions and behaviors. Second, a favorable attitude towards a technology is generally associated with a process of acquiring deep knowledge in the field of that technology, which can challenge competing attitudes such as a favorable attitude towards chemical fertilizers. This issue is important in the sense that in many agricultural societies, the use of green manures is not yet widespread. Therefore, strengthening the attitude towards green manures can help to increase the intention to use green manures by using knowledge development. Third, people with similar attitudes usually have more influence on each other. Therefore, improving the attitude towards green manure in a spectrum of agricultural society can lead to wider social changes in this field through the process of interpersonal interaction. Attitude has always been a key variable in encouraging pro-environmental intentions and behaviors. The present study also confirmed this evidence. In this regard, the policy-makers, managers, and decision-makers of technology development and transfer programs are recommended to create a favorable attitude towards the technologies in the target community before implementing technology transfer programs. Different methods and solutions can be used to create a favorable attitude towards a technology (such as green manure). Informing farmers about the short-term and long-term economic and environmental benefits of using green manure is one of the main strategies that can play a key role in creating a favorable attitude towards it. Awareness of the negative consequences of not using green manure is the second strategy that can be applied to change attitudes. In other words, in this strategy, farmers have a favorable attitude towards green manure by being aware of the harms and rebound effects of using chemical fertilizers. Attitude changes created using these strategies can ultimately lead to an increase in intention towards using green manure.

Among the variables that directly affected intention towards using green manure, perceived behavioral control on using green manure is the third strongest predictor. Based on the results of SEM, this variable had a positive and significant effect on intention towards using green manure. This result is in line with the results of Adnan et al.^43^, Kumar^47^, and Yarimoglu and Gunay^50^. Perceived behavioral control generally refers to the perceived ease or difficulty of using a particular technology, such as green manure. The more difficult it is for farmers to use green manure, the less inclined they will be to use it. However, if they find it easy to use, they will be more inclined to use it. Based on this, it can be argued that perceived behavioral control on using green manure should be improved among farmers to encourage intention. It is suggested that internal and external stimuli be used to enhance perceived behavioral control on using green manure. Internal stimuli originate within the farmers themselves and are a kind of reward they give themselves. Self-caring, flexibility, and avoidance of self-comparison are among the internal stimuli that can help strengthen perceived behavioral control on using green manure. Self-care in the application of green manure technology, even if a farmer does not perform well compared to other farmers, can help him/her feel highly self-productive. Avoiding comparing yourself to farmers is also similar to self-caring strategy in terms of performance. Because in this strategy, farmers realize that everyone has a special capacity and ability to use different technologies such as green manure. Therefore, in many cases it is wrong to compare their performance with other farmers. Farmer flexibility more than self-caring and avoiding comparing him/herself with others can be effective in increasing their level of personality resistance and increase the ability to face any challenges or adversity in the field of using green manure technology. The fact is that criticism stays in people's minds longer than admiration, and sometimes becomes important emotional events that are not easy to forget. Imagine that every time farmers face negative criticism, they lose all confidence in a short period of time. Thus, trying to increase realistic performance and accepting that not all criticisms are necessarily correct can be effective in forming a flexible personality in farmers who tend to use green manure. Planners, decision-makers, and practitioners of behavioral change programs for green manure adoption can use these strategies to achieve goals quickly. Of course, it should be mentioned that more successful implementation of these strategies requires the use of external incentives such as government support and financial facilities.

According to the results, trialability of green manure was the fourth variable that had a direct positive and significant effect on intention towards using green manure. This result shows that with increasing trialability of green manure technology, farmers are more willing to use it. This result has been supported by Bagheri et al.^48^ and Haji et al.^59^. Trialability helps increase farmers' confidence in the positive results of using green manure. As a result, in the midterm, it can lead to the development of the adoption of this technology. In this regard, it is necessary for technology disseminators to prove the trialability of green manure technology to farmers by creating demonstration farms at the micro level. In addition, it is suggested that researchers conduct field experiments on the effect of using green manures on rice yield. Of course, it should be mentioned that focusing research on experiments in this field requires the support of policy-makers and decision-makers. Because, if the development of sustainable methods of rice production is not included in the policies, it cannot be expected that organized and applied research can be done in this field. Addressing these recommendations allows farmers to use technology on their own farms at the micro level at the second stage. As a result, in a short period of time, they become confident enough about the results of using green manure and use it extensively on their farms.

The results of bootstrap analysis showed that farmers' subjective norms in the field of green manure application have an indirect, positive, and significant effect on intention towards using green manure. The direct effect of subjective norms on intention has been confirmed in many research studies (see^43,45,50,64^). However, in this study, an attempt was made to investigate the mediating role of trialability and moral norms in the relationship between subjective norms and the intention to use green manure. Due to the significant positive and indirect effect of subjective norms on intention towards using green manure, it is recommended that the implementers of behavioral change programs use control interactions as an effective tool to facilitate and encourage the intention to use green manure. To this end, efforts should be made to identify individuals who have intellectual and ideological influence in the agricultural community. Then these influential people will be convinced that using green manure technology can have many benefits for the agricultural community. By using green manure by leaders, other farmers, who generally follow thought leaders in action, will be more inclined to use it. Because, if they do not use green manure, they will feel that their thought leaders do not approve their work. This social pressure, which acts as a behavioral controller, has two key effects that indirectly improve the intention to use green manure. First, the use of green manure by thought leaders demonstrates the trialability of technology for farmers. Second, the actions of thought leaders in the Iranian agricultural community are generally the basis for moral judgment. In other words, if thought leaders use or approve of green manure, it means that they also consider the use of this technology to be a moral act. Thus, subjective norms as a social controller indirectly (mediated by moral norms and trialability) affect intention.

## Conclusion and future research pathways

In balance, the present study resulted in five key conclusions that can be used by planners, decision-makers, and field practitioners of agricultural technology dissemination to encourage the intention to use green manure. First, moral norms of green manure, attitude towards green manure, perceived behavioral control on using green manure, and trialability of green manure are four key variables that have direct, positive, and significant effects on intention towards using green manure. Second, moral norms mediate the effect of subjective norms on intention. Third, trialability also mediates the effect of subjective norms on intention towards using green manure. The second and third conclusions were one of the most important original contributions of the present study that had not been examined in previous studies. Fourth, the PLS-based SEM results demonstrated that the combination of TAM and IDT in the form of an integrated model is a reliable and valid model to encourage the intention to use green manure even among farmers outside the scope of this study. From a practical point of view, these four conclusions can play a decisive role in facilitating and encouraging behavioral change and the intention to use green manure in agricultural communities.

There were five main limitations in the present study, the description of which can both clarify the process of the present study and pave the way for further research in this field. First, in the present study, the self-reporting system was used to collect information on the intention to use green manure and its determinants. However, future researchers can use reference data to test the accuracy of these results. Although this reference data is not available in some countries, such as Iran, it may be present in some developed countries that are seriously analyzing their ecological footprints. Second, this study was conducted only in Iran. Although sampling has been done scientifically and the results of the data-model fit also indicate the reliability of the model, but the repetition of this research using cross-validation in other countries can strengthen the stability of the results and model in different spatial and temporal scopes. Therefore, the generalizability of research results increases. Third, the conceptual framework of the present study is derived from a combination of TPB and IDT. However, in these two frameworks, a limited number of variables are considered as predictors of the intention to use green manure. Therefore, we claim that the framework presented in this research is open for further development and future researchers can extend this framework by adding other socio-economic variables. For example, circular premium is one of the most important factors that might activate the intention of farmers to use green manure. Circular premium emphasizes the importance of the fact that the variables related to the demand chain can also have a significant effect on strengthening the intention of producers to use green manure. Fourth, in this study, the target population was rice farmers. Future researchers could go a step further and explore the intention to use green manure among other farmers. Fifth, the data in this paper were collected during the Covid-19 epidemic. The data collection team and the respondents had to use masks throughout the data collection process and observe social distance. This may have influenced the responses of some respondents.

## Supplementary Information

Supplementary Information 1.

Supplementary Information 2.

## Data Availability

The datasets used and/or analysed during the current study are available from the corresponding author on reasonable request.
