# Supplementary material for: Encouraging adoption of green manure technology to produce clean rice product
Source: Sci Rep. 2023 May 29;13:8690. doi: 10.1038/s41598-023-35964-1 (PMC10226031; doi:10.1038/s41598-023-35964-1)
Supplement: Supplementary file 2 — Supplementary Information 2. [file 41598_2023_35964_MOESM2_ESM.docx]

| **Supplementary table of legends for the data** | |
| --- | --- |
| **Legend in the datasheet file** | **Full name of the variable** |
| Intention | Intention towards using green manure |
| PUGF | Perceived behavioral control on using green manure |
| MNGF | Moral norms of green manure |
| AGF | Attitude towards green manure |
| TGF | Trialability of green manure |
| SNAGF | Subjective norms towards application of green manure |
